# Neurogenic Stunned Myocardium Following an Attempt to Pass a Drug Test

**DOI:** 10.7759/cureus.18318

**Published:** 2021-09-27

**Authors:** Zachary Illg, Aya Dabaja, Laura Garcia, Nicole Adams, Satheesh Gunaga

**Affiliations:** 1 Emergency Medicine, Henry Ford Wyandotte, Wyandotte, USA; 2 Emergency Medicine, Emory University, Atlanta, USA; 3 Emergency Medicine, Ascension Sacred Heart Pensacola, Pensacola, USA

**Keywords:** drug screen, extracorporeal membrane oxygenation, cerebral edema, acute hyponatremia, neurogenic stunned myocardium

## Abstract

Ingestion of a large volume of free water or other hypotonic solution can cause acute hyponatremia, leading to multiorgan dysfunction. Individuals may attempt to generate a false-negative urine drug screen through increased free water consumption leading to acute hyponatremia requiring emergency medical care. We present the case of a 19-year-old male who presented to a community emergency department for altered mental status after an attempt to generate a false-negative urine drug screen. He ingested a large volume of free water and multiple detoxification solutions, causing acute hyponatremia with resultant cerebral edema and neurogenic stunned myocardium. He required extracorporeal membrane oxygenation therapy with complete recovery of neurologic and cardiac function. Acute hyponatremia from excess free water consumption is a well-documented phenomenon that all emergency providers should be aware of. Prompt identification and management of acute hyponatremia are essential to prevent potentially severe, devastating sequelae, including cerebral edema and cardiopulmonary failure.In addition, extracorporeal membrane oxygenation may be considered in patients with cardiopulmonary failure in the setting of reversible cardiomyopathy, as evidenced in our case.

## Introduction

Symptoms of hyponatremia include headache, confusion, nausea, and in severe cases, seizures and coma [1]. Polydipsia leading to hyponatremia is a well-documented phenomenon [2-7]. Individuals may attempt to generate a false-negative urine drug screen through methods including detoxification solutions and increased free water consumption [8]. We present the case of a 19-year-old male who ingested various liquids in an attempt to pass a drug test. He subsequently developed cerebral edema, neurogenic stunned myocardium (NSM), and cardiopulmonary failure requiring extracorporeal membrane oxygenation (ECMO).

## Case presentation

A 19-year-old male with a history of substance use disorder presented to a community emergency department (ED) for altered mental status. The patient was accompanied by his father, who reported that the patient had a court date later that day where he would be required to undergo drug testing. The patient had reportedly used marijuana and cocaine the prior evening. The patient’s father reported that throughout the day, the patient drank three bottles of an unknown detoxifying solution, water, “a few shots” of hydrogen peroxide, and had two drinks consisting of 8 oz of water and 20 drops of hydrogen peroxide in the first and 8 oz of water and 10 drops of hydrogen peroxide in the second. Approximately one hour before arrival to the ED, the patient began to experience a headache, numbness in his arms and legs, nausea, vomiting, and confusion.

Upon arrival to the ED, the patient was agitated and exhibited incomprehensible speech. He was diaphoretic and had dry oral mucosa. His pupils were equal and reactive, and he had no appreciable focal neurologic deficits. Initial vital signs were grossly unremarkable. The patient was subsequently intubated for airway protection and to facilitate emergent imaging.

Laboratory testing was significant for serum sodium of 122 mmol/L, potassium of 2.7 mmol/L, and chloride of 88 mmol/L. His lactic acid was elevated at 5.8 mmol/L, and an arterial blood gas revealed a pH of 7.27 with an HCO3- of 20.1 mmol/L and a CO2 of 45.5 mmHg. ALT and AST were mildly elevated at 47 IU/L and 82 IU/L, respectively. Urine drug testing was positive for cannabinoids and benzodiazepines. A computerized tomography (CT) scan of the head, chest, abdomen, and pelvis was obtained. CT imaging of the head was remarkable for apparent obliteration of the basal cisterns concerning early global edema.

**Figure 1:**
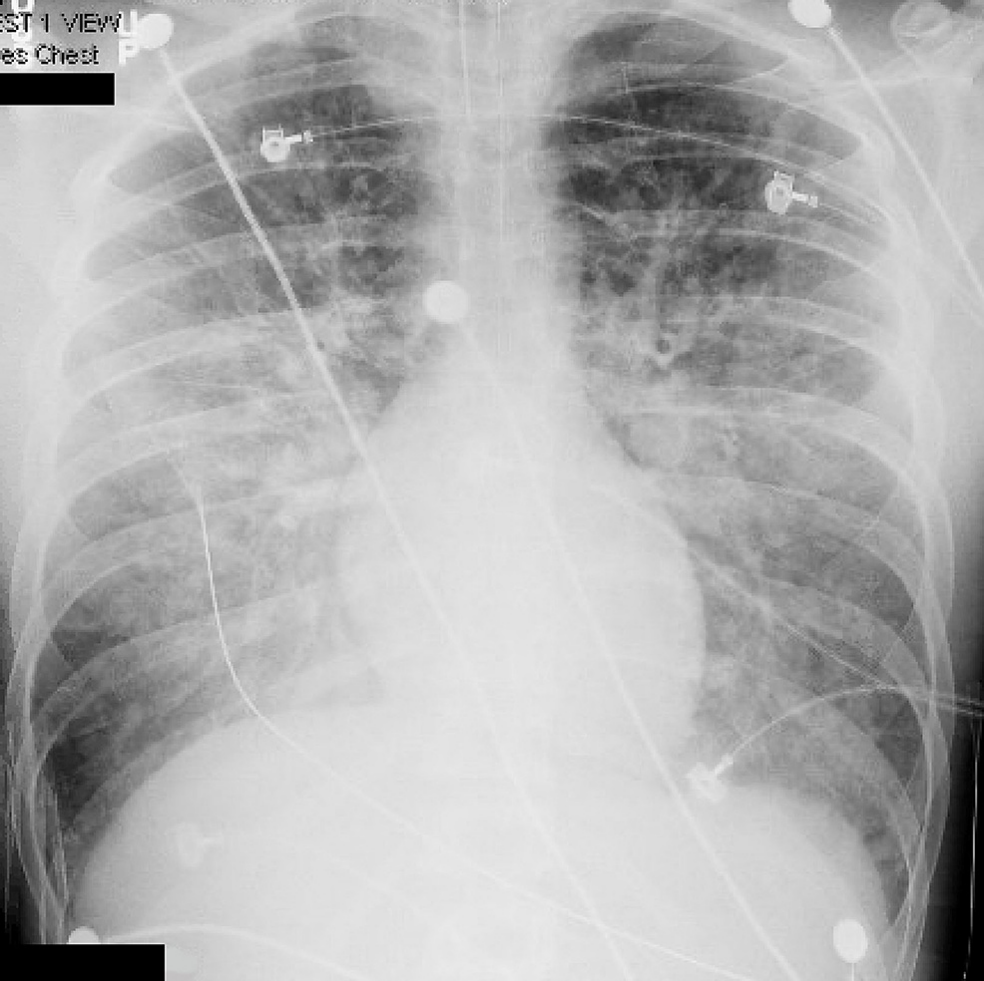
Chest x-ray showing pulmonary edema

Given imaging concerning global edema in the setting of hyponatremia, the patient was treated with hypertonic saline. Despite this therapy, the patient’s condition deteriorated, and he began to exhibit posturing with fixed, dilated pupils. The patient was subsequently treated with mannitol. Additionally, the patient became hypoxic on the ventilator with increasing positive end-expiratory pressure (PEEP) requirements. The decision was made to transfer the patient to a tertiary care hospital for neurointensive care. While hospitalized, the patient was noted to have a worsening serum sodium concentration of 115 mmol/L and was treated with additional hypertonic saline. The patient also had refractory hypoxia despite sedation, neuromuscular blockade, and increasing PEEP requirements. Chest imaging was concerning for pulmonary edema (Figure 1). On the second day of hospitalization, the patient had elevated troponin I level with a peak value of 5,170 ng/L, and an echocardiogram revealed that the patient had an ejection fraction (EF) of 36% with global hypokinesis. The decision was made to place the patient on venovenous ECMO for refractory hypoxia in the setting of cardiopulmonary failure.

The patient’s hyponatremia was aggressively managed, and his hemodynamic status improved upon initiation of ECMO. The patient’s clinical condition continued to improve, ECMO was discontinued 24 hours after initiation, and approximately six days after presentation to the ED, the patient was discharged home. At discharge, the patient was noted to be alert and oriented, without focal deficits. Approximately three months following discharge, the patient had repeat echocardiography performed, which showed recovery of cardiac function with an EF of 60%.

## Discussion

Acute hyponatremia can have rapid and severe consequences without prompt identification and proper management. Hyponatremia is defined by a serum sodium concentration <135 mmol/L [9]. Hyponatremia results in an osmotic gradient that shifts water from extracellular to intracellular compartments, which causes cerebral edema when it occurs in the brain [10]. Acute hyponatremia occurs in less than 48 hours, and the focus should be on prompt identification and treatment with 3% hypertonic saline if neurologic symptoms are present [11]. A sodium correction of 4-6 mmol/L has been shown to be effective in treating hyponatremic patients with seizures or coma [12].

**Figure 2:**
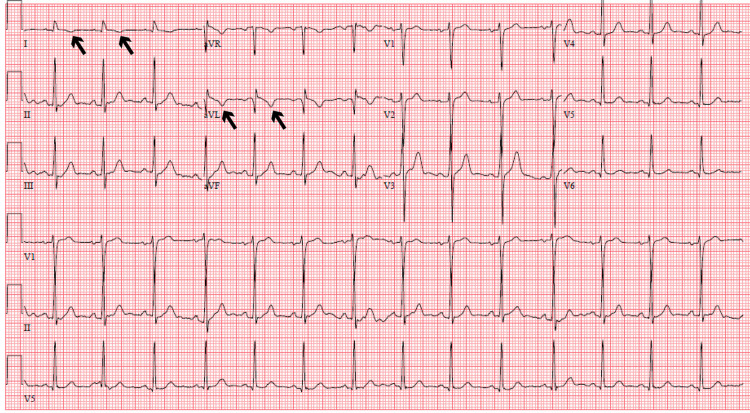
Electrocardiogram showing T-wave inversions in lead I and aVL

Consideration was given to hydrogen peroxide poisoning contributing to the patient’s clinical presentation as he had reportedly consumed an unknown quantity of hydrogen peroxide. However, the lack of cerebral or portal venous gas on CT imaging made this unlikely. Given cerebral edema in the setting of hyponatremia, the patient was treated aggressively with hypertonic saline but continued to deteriorate. He developed heart failure with reduced ejection fraction, which is thought to be secondary to NSM, which can be seen in neurologic injury. NSM occurs following a surge in catecholamine release after neurologic injury with the subsequent development of reversible stress cardiomyopathy [13]. There are no specific diagnostic criteria for NSM, but new echocardiographic abnormalities in the setting of neurologic insult are a primary diagnostic feature [14]. However, abnormal cardiac biomarkers and electrocardiographic (ECG) findings are often present in NSM [15]. The patient in our case had global hypokinesis on echocardiography, T-wave inversions on ECG (Figure 2), and elevated cardiac biomarkers with no prior history of heart disease. He had a complete recovery of cardiac function on follow-up imaging. A similar case has been documented in the literature in which a patient with a urinary tract infection drank a large volume of free water with resultant hyponatremia, neurologic injury, and NSM [5].

## Conclusions

Acute hyponatremia from excess free water consumption is a well-documented phenomenon that all emergency providers should be aware of, given its potential for devastating neurologic outcomes. Hyponatremia has the potential to cause cerebral edema with a resultant catecholamine surge causing reversible stress cardiomyopathy. ECMO may be a consideration in patients with cardiopulmonary failure in the setting of reversible cardiomyopathy, as evidenced in our case.
